# A versatile inducible plant model system for dissecting p53 function and discovering novel interaction networks

**DOI:** 10.1016/j.isci.2026.116775

**Published:** 2026-07-14

**Authors:** Yuqi Li, Shuyuan Wang, Steven Bell, Joanna Edwards, Ahmed Alboraey, Xiaopeng Wen, Chunli Chen, Patricia Muller, Miguel de Lucas

**Affiliations:** 1 Key Laboratory of Plant Resource Conservation and Germplasm Innovation in Mountainous Region (Ministry of Education), Institute of Agro-Bioengineering, College of Life Science, Guizhou University, Guiyang 550025, China; 2 Biosciences Department, Durham University, DH1 3LE Durham, UK; 3 National Key Laboratory for Germplasm Innovation and Utilization for Fruit and Vegetable Horticultural Crops, Hubei Hongshan Laboratory, Wuhan, Hubei 430070, China; 4 College of Life Science and Technology, Huazhong Agricultural University, Wuhan, Hubei 430070, China

**Keywords:** arabidopsis, p53, developmental cell death, apoptosis

## Abstract

p53, a key tumor suppressor in animals, prevents DNA damage by arresting the cell cycle, activating repair, or inducing cell death. Mutations in p53 are found in over half of human cancers, making it vital for cancer research. To expand p53 research, we engineered *Arabidopsis thaliana* to express p53 under an inducible promoter, allowing precise control of its activity. The plant-produced p53 shares certain characteristics with mammalian p53 in molecular traits and subcellular localization. Interestingly, its overexpression triggers cell-cycle arrest followed by a developmental programmed cell death-like response. Copper ions also suppress p53 activity in this system. This tool also enables investigation of tissue regeneration using cell type-specific promoters, offering insights into p53’s broader roles and the regulation of cell death in plants.

## Highlights

• Human p53 in *Arabidopsis* recapitulates mammalian molecular traits and interactions
• p53 expression in plants triggers G1 arrest and a dPCD-like response
• Copper ions suppress p53 activity in plants, as in mammalian cells
• Cell type-specific p53 induction is a tool to study organ regeneration

## Graphical abstract

**Figure:**
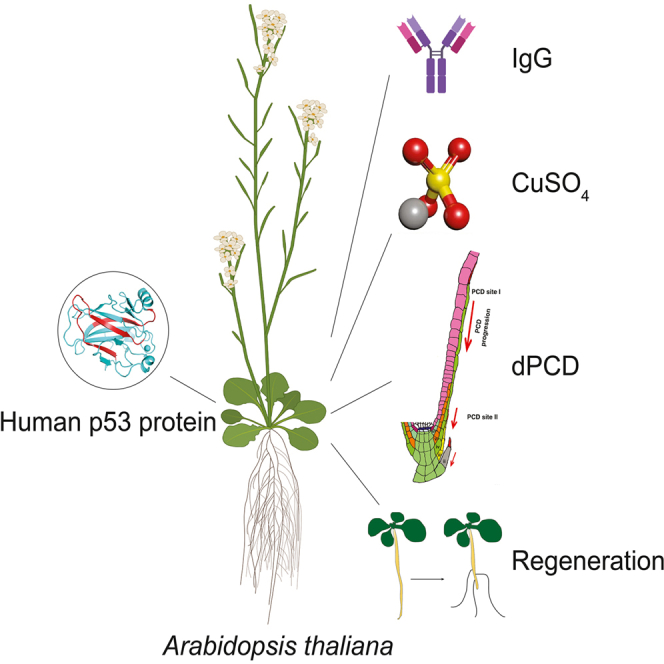

## Teaser

Biological sciences

## Introduction

In animal systems p53, is a tumor suppressor and transcription factor that regulates many genes upon cellular stress. Stresses that cause DNA damage lead to p53 stabilization and activation via DNA damage sensing proteins such as ATR and ATM.^1^^,^^2^ In response to small amounts of DNA damage, p53 will regulate p21 to induce cell-cycle arrest and promote expression of DNA repair enzymes. However, in response to large amounts of DNA damage, p53 will trigger apoptosis via regulation of proteins such as Bax and Puma.^3^ p53 may also regulate other types of cell death, including ferroptosis, entosis, pyroptosis, and cuproptosis,^4^ but the genes involved in these processes and the mechanisms by which p53 regulates them requires further study. p53 mutations occur in over 50% of human cancers and lead to either loss of p53 gene expression or expression of p53 mutant versions that often show perturbed activity.^5^ Many of such mutant p53 proteins are transcriptionally inactive as DNA-binding motifs are disrupted or because they lead to unfolded forms of p53 protein. Correct folding of p53 is dependent on zinc ion binding^6^ and other metal ions, including copper. Copper has been shown to interfere with p53 binding to zinc, resulting in p53 unfolding and loss of its transcriptional activity.^7^^,^^8^ Despite p53 being one of the most studied proteins, its extensive effects on transcription and the resulting apoptosis program often obscure the study of its more subtle functions. We present a new p53-inducible plant model system to study p53 activity. As many of the apoptotic machinery important in human cells are not present in plants, we believe such subtle intrinsic functions of p53 may be better studied in plants.

Traditional p53 research relies on patient biopsies, transgenic animal models, cancer cell cultures, and cell transfection experiments, which can be technically challenging and could restrict it to a limited number of laboratories. To expand access to p53 research, we developed transgenic *Arabidopsis thaliana* plants capable of producing p53 under an inducible promoter system. This approach enables precise temporal and spatial control of p53 expression for studying its activity. We also took advantage of the highly stereotypical and simple cellular structure of the Arabidopsis root to study the influence of p53 expression on cellular behaviors. This structure is composed of single cylindrical cell layers of epidermis, cortex, and endodermis cells that surround the vascular tissue^9^ (Figure S1). Moreover, its primary growth results from cell division and elongation processes occurring in the distal end, which contains actively dividing cells.^9^

The p53 protein produced in *Arabidopsis* exhibits molecular characteristics analogous to its mammalian counterpart, including comparable molecular weight, antibody recognition patterns, and subcellular localization. Most notably, when overexpressed, plant-produced p53 triggers cell-cycle arrest and cell death. Different mechanisms underlie cell death in plants. Environmental programmed cell death (ePCD) occurs in response to abiotic stresses such as temperature or radiation^10^ or biotic agents such as pathogens^11^; while developmental programmed cell death (dPCD) is intrinsic to specific cell differentiation programs. For example, dPCD occurs during xylem cell differentiation,^12^ lateral root cap (LRC) development,^13^ or the maturation of the anther tapetum.^14^ We demonstrate that p53 expression in plants induces a developmental programmed cell death-like (dPCD-like) process analogous to its apoptotic activity in mammalian cells. Moreover, the cell death function of plant expressed p53 can be inhibited by copper ions, similar to what occurs in animal cells. Investigating how p53 induction leads to cell death in plants could uncover previously unidentified regulators and may infer shared mechanisms across kingdoms. This is particularly intriguing given that plant genomes appear to lack sequence homologues of the key apoptotic regulators found in animals.^15^

Plants have an extraordinary capacity for organ regeneration, driven by the ability of differentiated cells to revert to a pluripotent state and acquire new developmental cell fates. Deciphering the hierarchical sequence of cellular and molecular events underlying this process remains a central question in developmental biology, with significant implications for agricultural innovation.^16^ Expanding the toolkit for precise characterization of these regenerative mechanisms is therefore relevant. In this study, we also validate the use of p53-induced cell death as an experimental strategy to probe root tissue regeneration and confirm the role of specific cell types involved in this dynamic process.

## Results

### Heterologous expression of human tumor suppressor *p53* in *Arabidopsis*

**Figure 1:**
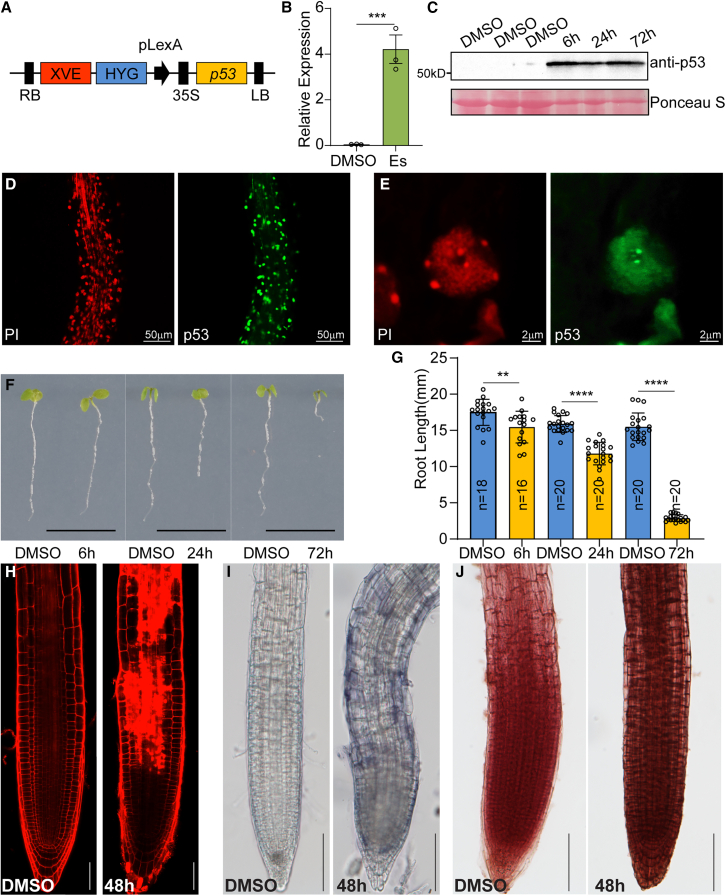
Systemic induction of human p53 expression in *Arabidopsis* (A) Construction of the inducible expression vector with p53 by the XVE vector. LB, left border; RB, right border; HYG, hygromycin resistance gene; 35S, cauliflower mosaic virus minimal 35S promoter. (B) The transcript level of p53 in 5-day-old seedlings after treated with DMSO or β-estradiol for 24 h. (C) The protein level of p53 in 5-day-old seedlings after treated with DMSO or β-estradiol for 6, 24, and 72 h, respectively. (D and E) p53 localization in 5-day-old seedlings root. Propidium iodide staining showing the location of the nuclei (left panel) and Alexa Fluor 488 signal indicative of p53 location (right panel). Scale bars, 50 μm (D) and 2 μm (E). (F) Representative 5-day-old root seedlings of mock, β-estradiol treated (6, 24, and 72 h) XVE:p53. Scale bars, 0.5 cm. (G) Primary root length measurements. N is number of seedlings, Bar plots represent the mean ± SD and asterisks indicate significant differences between samples (∗, <0.05; ∗∗, *p* < 0.01, ∗∗∗, *p* < 0.001, ∗∗∗∗, *p* < 0.0001 by Student’s *t* test, ns is no significant). (H) Representative confocal images of 5-day-old XVE:p53 roots treated with DMSO or 10 μM estradiol, stained with propidium iodide. Scale bars, 50 μm. (I) Trypan blue staining for the detection of cell death in XVE:p53 roots treated with DMSO and estradiol. Scale bars, 200 μm. (J) Microscopic images exhibit the effects of corresponding chemical treatments on the accumulation of H_2_O_2_ (detected by DAB staining). Scale bars, 200 μm.

To express p53 in plant cells, we inserted the human p53 coding sequence (NCBI Gene ID: 7157) in the pER8 plasmid, which allows for p53 induction ubiquitously upon application of β-estradiol (Figure 1A).^17^ Transgenic plants were generated and tested for induced p53 at the transcriptional (RT-qPCR) and protein level (western blot) using p53-specific primers and antibodies respectively. p53 transcripts were induced more than 4-fold after 24 h of induction and the p53 protein was detected as a single band of ∼53 KD, as expected for human p53, confirming that our XVE:p53 transgenic plants can induce the expression of the p53 gene and produce a stable full-length protein (Figures 1B and 1C).

In human cells, p53 predominantly localizes to the nucleus.^18^ Consequently, we examined the subcellular localization of the plant-expressed p53 through whole-mount immunofluorescence using an anti-human p53 antibody and revealed that plant-expressed p53 localizes exclusively in the nucleus (Figures 1D and 1E).

### p53 overexpression inhibits root growth in *Arabidopsis*

*Arabidopsis* roots are ideal systems to study cellular behaviors.^9^ Consequently, we studied the effect of p53 on the behavior of the *Arabidopsis* primary root by measuring its influence on root growth after 6, 24, and 72 h of p53 induction. We observed that expressing p53 strongly inhibited root growth, with the inhibition becoming more severe with longer inductions (Figures 1F and 1G).

Inhibition of primary root growth can result from perturbations in mitotic activity of cells in the meristem, from failures in cell elongation of the cells in the elongation and differentiation zones, or from cell death.^19^ To investigate if p53 expression regulated these processes, we used propidium iodide (PI) staining in combination with confocal microscopy. PI stains plant cell walls but also diffuses into subcellular compartments and binds DNA when plasma membrane integrity is compromised.^20^ This dual property of PI has been extensively used to track organ architecture and cell death. When estradiol was applied on 5-day-old seedlings for 24 h, we found that, in contrast to mock-treated seedlings, PI staining produced a strong signal inside multiple cells of the root, which is indicative of cell death (Figures 1H and S2C). This result was somewhat unexpected, as plant genomes do not encode clear homologues for the principal human apoptotic regulators,^21^ especially those regulated by p53.^22^ Consequently, to validate that p53 induction triggers cell death in *Arabidopsis* cells we used trypan blue and DAB (3,3′-diaminobenzidine) dyes. The former penetrates damaged cell membranes staining dead cells in blue, while living cells remain unstained.^23^ As observed with PI, roots expressing p53 showed high numbers of cells stained with trypan blue, while control cells remained unstained (Figures 1I and S2D). Damaged or stressed cells tend to accumulate H_2_O_2_ due to failure in the cellular antioxidant defense system. In the presence of H_2_O_2_, DAB (3,3′-diaminobenzidine) is oxidized, producing a brown polymer that precipitates and can be visualized under a microscope.^24^ The number of brown aggregates in the roots expressing p53 increased compared to the control (Figures 1J and S2E). Altogether, our results confirm that p53 expression in plant cells triggers cell death, resulting in severe root growth arrest.

### p53 overexpression activates the expression developmental cell death genes

To gain further insights into the cell death mechanisms occurring upon p53 induction, we examined the expression of a series of marker genes involved in both dPCD and ePCD upon p53 induction using RT-qPCR.^25^ For dPCD analysis we used *CYSTEINE ENDOPEPTIDASE* (*CEP1*), *RIBONUCLEASE 3* (*RNS3*), *BIFUNCTIONAL NUCLEASE1* (*BFN1*), *PUTATIVE ASPARTIC PROTEASE A3* (*PASPA3*), and *DUF679 MEMBRANE PROTEIN 4* (*DMP4*). CEP1 is a papain-type cysteine protease involved in tapetum dPCD during pollen development.^26^ It is also expressed in dying LRC cells, which also undergo dPCD as part of their differentiation process.^27^ RNS3 degrades cellular RNA in senescing tissues such as petals, the outer layers of the anthers and differentiating seed coat.^28^ BFN1 functions in chromatin breakdown during LRC PCD and tracheary element dPCD.^29^^,^^30^ PASPA3 functions in vacuole collapse and protein degradation during dPCD.^31^ *DMP4* expression is tightly associated with various dPCD processes, including xylem differentiation and endosperm breakdown. It is often used alongside *PASPA3* to identify and confirm the onset of cell death in specific tissues.^25^ For the analysis of ePCD we used *MYB30*, *PATHOGENESIS-RELATED GENE 1* (*PR1*), and *METACASPASE 1* (*MC1*). MYB30 is a R2R3-TYPE MYB transcription factor which expression is induced by pathogen attack (especially *Pseudomonas syringae*), the presence of ROS and salicylic acid (SA).^32^ PR1 is a hallmark of systemic acquired resistance (SAR) and the local hypersensitive response (HR), but it does not directly cause cell death. However, PR1 expression correlates with cell regions during pathogen attack.^33^ MC1 is a positive regulator of pathogen-induced PCD that promotes the hypersensitive response (HR) after pathogen detection.^34^

**Figure 2:**
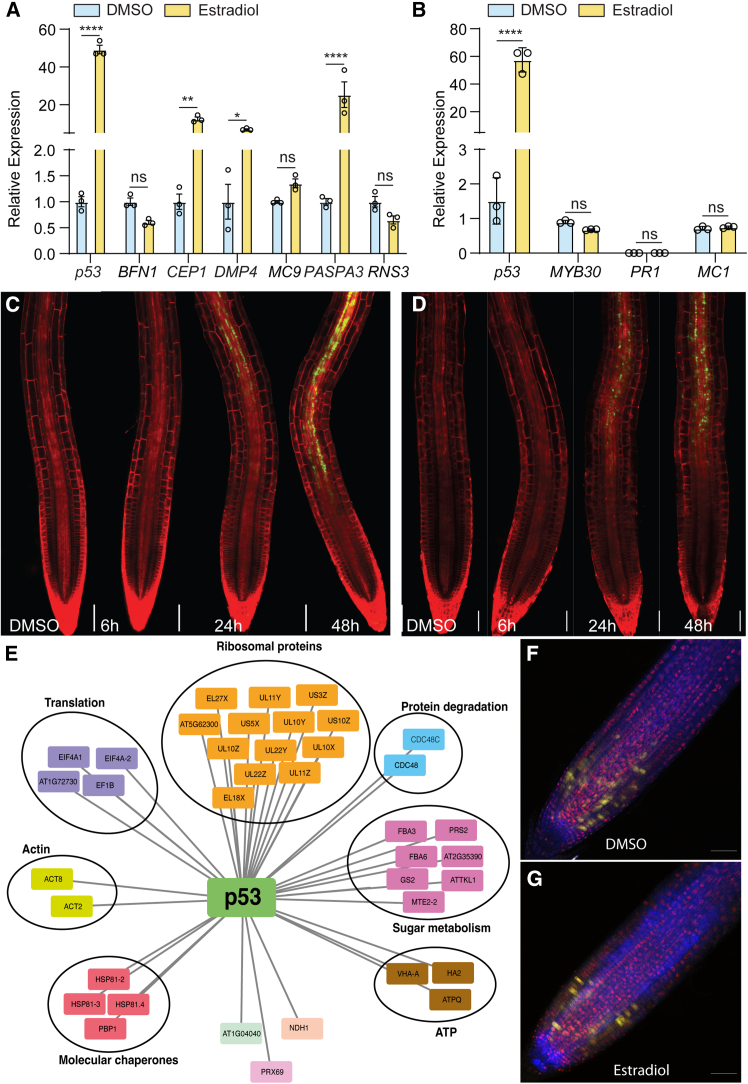
Developmental cell death-marker genes are induced by p53 (A) Transcript levels of developmental programmed cell death marker genes in XVE:p53 root after DMSO and β-estradiol treatment. (B) Transcript levels of environmental programmed cell death marker genes in XVE:p53 root after DMSO and β-estradiol treatment. Plots represent the mean ± SD and asterisks indicate significant differences between samples (∗, *p* < 0.05; ∗∗∗, *p* < 0.001, ∗∗∗∗, *p* < 0.0001 by Student’s *t* test, ns is no significant). (C and D) Representative confocal images of 5-day-old *pDMP4:H2B:3xVenus* (C) and *pPASPA3:H2B:3xVenus* (D) roots treated with DMSO and 10 μM estradiol for 6, 24, and 48 h in XVE:p53 background, scale bars, 50 μm. (E) p53 interactome in 3-week old XVE:p53 root. (F and G) Representative confocal images of 5-day-old PLACCI roots treated with DMSO (F) and 10 μM estradiol (G) for 24 h in XVE:p53 background, scale bars, 50 μm.

Among the five dPCD markers selected, we observed upregulation of *PASPA3*, *DMP4*, and *CEP1* after 24 h of p53 induction (Figure 2A). By contrast, we did not observe induction of any ePCD markers (Figure 2B). This suggests that p53 expression in plant cells induces an intrinsic programmed cell death that resembles those occurring during the differentiation of specific cell types. To further investigate the induction of dPCD markers by p53 in *Arabidopsis* roots, we generated fluorescent *DMP4* and *PASPA3* transcriptional reporters (*pDMP4:H2B:3xVENUS* and *pPASPA3: H2B:3xVENUS)* in the background of the p53 conditional expression line. Activation of both *DMP4* and *PASPA3* promoters was observed 24 h after p53 induction (Figures 2C and 2D). Interestingly, the signals were predominantly detected in the vascular cells in the elongation and differentiation zones of the root and excluded from the meristematic zones.

### Proteomic profiling reveals functional recapitulation of p53 interactions in plants

With the aim of revealing potential regulatory partners shared with mammalian p53 or unique to the plant context, that could explain p53 activity in plants, we performed p53-immunoprecipitation in combination with mass spectrometry (IP-MS) on 3-week-old XVE:p53 root cultures treated with DMSO (control) or 10 μM β-estradiol (inducer) for 24 h. We defined positive p53-interacting proteins based on peptide coverage and appearing in at least two of the three β-estradiol biological replicates, but absent in any of the three control biological replicates. Following this criterion, a list of 38 individual proteins was considered *bona-fide* p53 interacting partners in plants (Data S1). We next performed gene ontology analysis^35^ (Data S1) to identify in which processes p53 interactor’s function. Protein translation, gene expression and various fundamental metabolic processes were identified as enriched ontologies. Importantly, some of the interactors are well-known homologues of mammalian p53 interactors, such as glucose-6-phosphate dehydrogenase,^36^ translation elongation factors,^36^ the 90KD Heat shock protein (HSP90),^37^ the segregase and unfoldase cell division cycle 48 (CDC48, also referred as p97 or valosin containing protein VCP)^38^ or actin^39^ (Figure 2E), which suggest that p53 retains at least some of its functional interactions when expressed in this heterologous plant system.

### Mammalian p53-mediated cell-cycle arrest may be recapitulated in plants

Given that p53 is well established as a mediator of G1 cell-cycle arrest following DNA damage in animals,^40^^,^^41^ we asked whether its expression in plants might also cause arrest the mitotic cell cycle at G1. To test this, we crossed the XVE:p53 inducible line with the PlaCCI cell cycle reporter, which enables simultaneous visualization of all major cell cycle phases within a single image^42^ (G1 is marked by pCDT1A:CDT1A:CFP, S phase by pH3.1:H3.1:mCherry, late G2 by co-expression of pH3.1:H3.1:mCherry and pCYCB1;1-N-CYCB1;1:YFP, and M phase by co-expression of pH3.1:H3.1:mCherry and pCYCB1;1-CYCB1;1:YFP). Comparing PlaCCI fluorescence in XVE:p53 roots treated with mock or β-estradiol for 24 h, we observed an increase in the number of meristematic cells displaying CFP signal, indicative of G1 arrest (Figures 2F and 2G; Figures S3). Together, these results suggest that p53 expression is sufficient to promote G1 arrest in the plant root meristem.

### Copper ions perturb p53 activity in *Arabidopsis*

**Figure 3:**
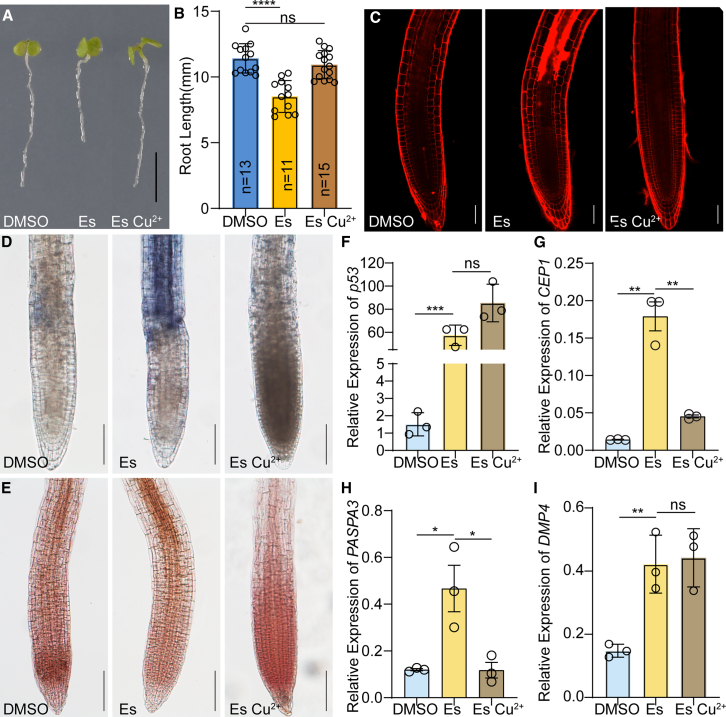
Copper inhibits p53 activity in plants (A) Representative 5-day-old XVE:p53 root seedlings, scale bars, 0.5 cm. (B) Primary root length measurements of 5-day-old XVE:p53 seedlings. N is the number of seedlings. Bar plots represent the mean ± SD and asterisks indicate significant differences between samples (∗∗∗∗, *p* < 0.0001, ns, no significant by Student’s *t* test). (C–E) Representative images of 5-day-old XVE:p53 roots treated with DMSO, 10 μM β-estradiol, or 10 μM β-estradiol supplemented with CuSO_4_ (5, 10, 25, and 50 μM)), from left to right. (C) Confocal images of roots stained with propidium iodide. Scale bars, 50 μm. (D) Trypan blue staining for the detection of cell death. Scale bars, 200 μm. (E) Accumulation of H_2_O_2_ detected by DAB staining. Scale bars, 200 μm. (F–I) Transcript levels of *p53* (F), *CEP1* (G), *PASPA3* (H), and *DMP4* (I) in XVE:p53 root. Plots represent the mean ± SD and asterisks indicate significant differences between samples (∗, *p* < 0.05; ∗∗, *p* < 0.01, ∗∗∗, *p* < 0.001 by Student’s *t* test, ns is no significant). All the 5-day-old XVE:p53 seedlings treated with DMSO (mock), 10 μM β-estradiol (Es) and 10 μM β-estradiol with 25 μM CuSO_4_ (Es Cu^2+^) for 24 h, respectively.

It has been shown that copper binding to p53 results in the loss of its function in human cells due to protein misfolding.^7^^,^^43^^,^^44^ To investigate if copper also inhibits p53 activity in plants, we transferred 4-day-old XVE:p53 seedlings to media containing different concentrations of copper sulfate (CuSO_4_) in the presence of β-estradiol. Differences in root growth were measured 24 h later. As for before, p53 overexpression led to a reduction of root growth in media without CuSO_4_. However, while the addition of CuSO_4_ did not show significant effect on the roots growing under 5 and 10 μM of CuSO_4_, when the concentration reached 25 μM, the short root phenotype was fully restored (Figures 3A and 3B; Figures S4A and S4B). To confirm that the recovery of root growth is not due to reduced p53 induction. In CuSO_4_ treated roots, we tested p53 expression upon induction through RT-qPCR and western blot and observed that it was comparable to the levels observed in the control plate (Figures 3F and S4).

To further validate that copper inhibited p53 activity, we studied the cell death markers PI, trypan blue and DAB in roots that have been treated with CuSO_4_ during p53 induction. Cells expressing p53 in the absence of CuSO_4_ incorporated all three dyes, consistent with p53 activity. However, the plants growing on concentrations of CuSO_4_ higher than 25 μM showed a reduction in the incorporation of the three markers, suggesting an inhibition of p53 function (Figures 3C–3E; Figures S4C and S4H). Next, we analyzed the behavior of the dPCD molecular markers that appeared to be induced after p53 induction (*CEP1*, *PASPA3*, and *DMP4*) through RT-qPCR. Interestingly, Cu^2+^ treatment abolished the induction of *CEP1* and *PASPA3* observed in p53 overexpressing cells (Figures 3G and 3H). However, it did not inhibit *DMP4* expression (Figure 3I). Altogether, these results indicate that, as observed for mammalian cells, p53-dependent responses are attenuated by the presence of Cu^2+^ ions. However, the maintenance of *DMP4* induction after Cu^2+^ treatment suggests that not all components of p53 activity are inhibited by this metal.

### p53 activity in plant cells is cell autonomous

**Figure 4:**
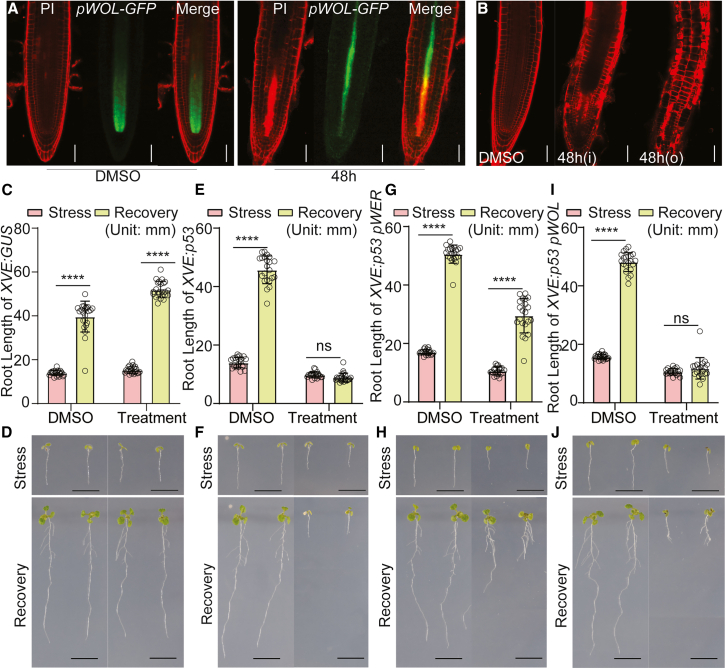
Cell autonomous activity of plant-expressed p53 (A) Representative confocal images of 5-day-old pWOL:XVE:p53 seedlings of DMSO and β-estradiol treatment for 2 days in pWOL:GFP background. All the roots stained with propidium iodide. Green fluorescent is Wooden leg signal. Scale bars, 50 μm. (B) Representative confocal images of 5-day-old pWER:XVE:p53 seedlings of DMSO and β-estradiol treatment for 2 days in Col-0 background. All the roots stained with propidium iodide. Scale bars, 50 μm. (C) and (D) Primary root length measurements (C) and representative images (D) of XVE:GUS seedlings after treatment with DMSO and estradiol for 2 days and after 5 days cultured with MS. Scale bars, 1 cm. (E) and (F) Primary root length measurements (E) and representative images (F) of XVE:p53 seedlings after treatment with DMSO and estradiol for 2 days and after 5 days cultured with MS medium. Scale bars, 1 cm. (G) and (H) Primary root length measurements(G) and representative images (H) of pWER:XVE:p53 seedlings after treatment with DMSO and estradiol for 2 days and after 5 days cultured with MS. Scale bars, 1 cm. (I) and (J) Primary root length measurements (I) and representative images (J) of pWOL:XVE:p53 seedlings after treatment with DMSO and estradiol for 2 days and after 5 days cultured with MS. Scale bars, 1 cm. N is number of seedlings, bar plots represent the mean ± SD and asterisks indicate significant differences between samples (∗∗∗∗, *p* < 0.0001 by Student’s *t* test, ns is no significant). Two-day-old seedlings (XVE:GUS, XVE:p53, pWER:XVE:p53, and pWOL:XVE:p53) were treated with DMSO or estradiol (treatment) for 3 days “stress” pictures are 5-day-old seedlings after treatment with DMSO or estradiol (treatment) for 3 days “recovery” pictures are 10-day-old seedlings, which are “stress” seedlings that move to MS medium for 5 days.

In human cells, p53-induced cell death is partially non-cell autonomous. This is, in part, because p53 induction of apoptosis causes the secretion of senescence factors that induce cell death in surrounding cells.^45^ Plant cells possess rigid cell walls, and their PCD mechanisms occur autonomously within each cell. To investigate whether p53 also exhibits non-cell autonomous behavior in plant cells, we restricted p53 expression to specific cell types. We replaced the pG10-90 ubiquitous promoter^46^ of the XVE system by the promoter of the *WOODENLEG* (p*WOL)* and *WEREWOLF* (p*WER*) genes (Figure S5). The *WOL* gene is specifically expressed in the cells of the vascular cylinder,^47^ while *WER* expression is specific to non-hair cells of the epidermis layer (atrichoblasts). The *pWOL:XVE:p53* and *pWER:XVE:p53* transgenes were inserted into the *pWOL:GFP* marker line and Col0 (wild type) backgrounds, respectively. For *pWOL*, we observed that after 2 days of β-estradiol induction, the PI staining penetrated only in cells belonging to the vascular tissue (Figure 4A). Similarly, in *pWER:XVE:p53* seedlings, the PI signal only penetrated the cells within the epidermis cell type (Figure 4B). This cell-specific induction of cell death indicates that, in *Arabidopsis* cells, p53 activity is cell autonomous.

Plants have an extraordinary regeneration potential. Root meristem excision analysis demonstrated that the pericycle cell type contributes to the regeneration of the grown tissue (endodermis and cortex cell layers) while endodermis cells regenerate the epidermis and lateral root cap cell types.^48^ These experiments proposed a model in which root meristem regeneration occurs outwards from the inner tissues. To evaluate if p53’s ability to induce cell death can be applied to investigate organ function, we compared the regeneration ability of roots expressing p53 under the control of the ubiquitous *pG10-90*, the epidermis-specific *pWER* and the vascular cylinder *pWOL* promoters for 2 days. Following this treatment, the seedlings were returned the control medium. To evaluate the regenerative growth potential associated with each promoter controlling p53 expression, root length measurements were taken as an indicator of the growth recovery.

The XVE:GUS expressing roots grew identically in the DMSO and estradiol treatments (Figures 4C and 4D). However, when p53 was induced for 2 days using the constitutive pG10-90 promoter, root growth did not recover and chlorosis, characteristic of death tissue, was observed along the entire plant (Figures 4E and 4F). The epidermis-specific induction of p53 using *pWER* promoter resulted in an initial reduction of growth that was restored few days after treatment. Interestingly, this treatment also induced lateral root formation (Figures 4G and 4H). By contrast, the induction of p53 in the vascular cylinder by *pWOL* caused a strong root growth arrest and the plants were unable to recover the root growth by day 5 (Figures 4I and 4J). These results are consistent with previous observations regarding root regeneration processes^48^ and demonstrate that p53 cell-type-specific induction serves as an alternative tool for investigating the contribution of distinct cell types to developmental processes.

## Discussion

Plants developed multicellularity independently of animals, however fundamental cell function mechanisms such as transcription, chromatin organization, and protein translation are still conserved across eukaryotic kingdoms. We have confirmed that the expression of p53 in plants generates a functional protein of identical molecular weight to p53 expressed in mammalian cells that is recognized by human p53-specific antibodies and localizes in the nucleus. Previous studies have also attempted to express functional p53 into plant cells using constitutive promoters. However, these approaches resulted in only modest senescence responses in mature leaf tissue.^49^ In contrast, our inducible promoter system demonstrated that p53 triggers pronounced cell death within 24 h of activation. This rapid and robust response suggests that constitutive p53 expression would likely prove embryonically lethal, with only plants carrying silenced or non-functional p53 variants surviving to reproductive maturity. The β-estradiol-inducible expression system overcomes these constraints by providing precise temporal and spatial control over p53 activity, offering a more effective tool for studying p53. Consistent with p53’s well-established role as a mediator of G1 cell-cycle arrest in mammals,^40^^,^^41^ induction of p53 expression in an inducible manner in the root meristem allowed us to reproduce p53 function in G1 cell-cycle arrest. p53 overexpression resulted in a significant accumulation of cells in G1 and a corresponding reduction in G2/M populations, as revealed by the PLACCI reporter. This recapitulation of cell cycle regulatory function further supports the ability of p53 to retain certain activities when expressed in plant cells.

The p53 interactome identified in plants includes several proteins with established roles as p53 partners in mammalian systems (Figure 2E), demonstrating that this heterologous context is sufficient to mirror key aspects of p53’s molecular function. We propose that this system could also facilitate the identification of novel p53-interacting partners. For example, plant p53 interacts with MITOCHONDRIAL PYRUVATE DEHYDROGENASE SUBUNIT 2-2 (MTE2-2), whose human homologue is dihydrolipoamide S-acetyltransferase (DLAT), the E2 catalytic subunit of the pyruvate dehydrogenase complex (PDC). DLAT catalyses the conversion of pyruvate to acetyl-CoA within the mitochondrial matrix, the committed step that feeds carbon derived from glycolysis into the TCA cycle.

In cancer cells, most glucose is converted to lactate even in the presence of oxygen, a phenomenon known as aerobic glycolysis or the Warburg effect.^50^ This metabolic reprogramming generates ATP more rapidly and provides biosynthetic precursors that support cell proliferation. p53 is a well-established suppressor of glycolysis, acting in part by transcriptionally activating TIGAR, which functions as a fructose-2,6-bisphosphatase, thereby lowering fructose-2,6-bisphosphate levels and reducing glycolytic flux. However, whether p53 directly regulates the activity of metabolic enzymes at the mitochondrial level remains poorly understood. In this context, a physical interaction between p53 and DLAT would be particularly significant, as DLAT has recently emerged as a central player in cuproptosis, a form of regulated cell death triggered by intracellular copper accumulation, in which copper directly binds lipoylated DLAT, causing its aggregation and proteotoxic stress.^51^ Furthermore, recent evidence implicates DLAT in promoting tumorigenesis adding further relevance to this potential interaction.^52^

There are many post-translational modifications (PTM) that affect the structure and dynamics of p53.^53^ Importantly, their mode of action is highly conserved across eukaryotes. For example, three E3 ubiquitin ligases, MDM2, Pirh2, and COP1, target human p53 for proteasome-mediated degradation.^54^^,^^55^ Some plant E3 ligases share sequence homology with these mammalian proteins: BRUTUS-LIKE proteins (BTLS1 and BTLS2) resemble Pirh2, while CONSTITUTIVE PHOTOMORPHOGENIC 1 (COP1) shows homology to its mammalian counterpart.^56^^,^^57^ Like their mammalian analogues, these plant E3 ligases also regulate transcription factor stability. Confirming p53 ubiquitination by *Arabidopsis* COP1 could demonstrate functional recapitulation of this regulatory pathway, supporting the potential use of plant-expressing p53 as an alternative system for studying the influence of PTMs on p53 function.

Moreover, as a zinc-binding protein, p53 activity is influenced by the presence of metals.^58^ Previous research demonstrated that copper ions alter p53 protein conformation and impair its function.^7^^,^^43^^,^^44^ Our research demonstrated that the activity of the plant-expressed p53 is also altered by copper, thus also supporting the use of plant-expressed p53 for investigating the role of metals in its function.

A critical component of p53 tumor suppressor activity is its ability to induce apoptosis. In mammals, this process occurs through three main pathways: the extrinsic pathway, the intrinsic pathway, and the endoplasmic reticulum stress pathway.^59^ p53 functions through activation of both intrinsic and extrinsic pathways, both of which involve the activity of cysteine proteases (caspases) that cleave proteins at aspartic acid residues.^22^ Instead of caspases, plants genomes encode for other endopeptidases that share little sequence homology with animal caspases. However, their protein structure and function resemble animal caspases.^21^^,^^59^ These are metacaspases and vacuolar processing enzymes (VPE).

The extrinsic apoptotic pathway is initiated when ligands bind to transmembrane death receptors, triggering recruitment of cytoplasmic adapter proteins that activate pro-caspases. This process leads to the formation of the death-inducing signaling complex (DISC), which subsequently activates executioner caspases and culminates in programmed cell death.^59^ The tumor suppressor p53 can induce the extrinsic pathway by transcriptionally upregulating ligands that bind to and activate these transmembrane death receptors.^22^ However, this pathway appears to be absent in plants, p53 activation of dPCD-like mechanisms may not occur through upregulation of dead receptor ligands.

The intrinsic pathway is activated in response to cellular damage and is tightly regulated by the Bcl-2 protein family, which comprises both pro-apoptotic and anti-apoptotic members. Following detection of apoptotic stimuli, pro-apoptotic Bcl-2 family proteins, including Bax and Bak, form pores in the outer mitochondrial membrane, facilitating the release of cytochrome *c* into the cytoplasm. Released cytochrome *c* associates with apoptotic protease-activating factor-1 (Apaf-1) to form the apoptosome complex, which activates initiator pro-caspases and triggers the downstream caspase cascade leading to cell death. p53 induces apoptosis, in part, through the induction of the expression of proapoptotic Bcl-2 proteins such as *Bax*, *Puma*, *Noxa* and *Bid*,^60^ and repression of the anti-apoptotic proteins BCL-xL and BCL-2^61^ There is evidence of the presence of anti-apoptotic Bcl-2-like proteins in plants, such as the Bax-inhibitor AtBI-1, which has been shown to inhibit *Bax* induced cell death in yeast.^62^ However, the presence of pro-apoptotic Bcl-2 proteins in plant genomes remains unclear. The finding that human p53 can induce cell death in plants and the existence of interactors with homologous functions those in animals reinforces the idea of the existence of functional proapoptotic Bcl-2 proteins antagonistic to AtBI-1. A better characterization of p53 activity in plant cells could help answer the intriguing but unresolved question of whether developmental cell death in animals and plants is evolutionarily conserved or the result of convergent evolution. Moreover, in addition to apoptosis, p53 has also been linked to various newly emerging types of regulated cell death such as necroptosis, ferroptosis, pyroptosis, and others.^4^ It’s role in these processes goes beyond being a tumor suppressor, and many of these processes overlap in human cells. Some of these types of cell death can be seen in plants.^63^ It will be interesting to further characterize p53-mediated regulated cell death in plant cells and investigate if we can shed a light on p53’s regulation in these types of cell death using differences as well as similarities between plants and humans.

Organ growth and development result from the coordinated interplay of cell division and differentiation processes within the organ’s cells. This coordination depends on effective communication between cells from different tissues. Cell function studies require approaches able of precisely modifying cells without perturbing the surrounding cellular environment and overall organ structure. Laser ablation techniques have been instrumental in advancing our understanding of organ function. For example, key developmental findings such as the principle that position within the organ, rather than lineage, defines cell identity have emerged from this approach.^64^^,^^65^ However, laser ablation only permits analysis of small sample numbers and requires specialized microscopy equipment that is not widely accessible. To overcome these limitations, researchers implemented the use of diphtheria toxin A chain (DTA), which functions by inhibiting elongation factor-2 (EF-2), an essential component required for protein synthesis, and consequently triggers cell death.^66^ Using cell-specific promoters, we demonstrated that p53 induction of cell death in plants is cell autonomous. This feature not only provides an advance for the study of p53 function in a cell-specific manner but also provides an alternative genetic approach for the study of the contribution of specific cell types to organ function.

The transient induction of p53 in epidermal cells caused localized cell death that was subsequently restored, likely through regenerative processes originating in the inner tissues. By contrast, induction of p53 in the inner tissues abolished organ regeneration, consistent with their role as the source of regenerative capacity. Notably, concomitant with epidermal cell death, a marked increase in lateral root formation was observed. It has been shown that cyclic programmed cell death in the lateral root cap defines the positions at which lateral roots subsequently develop, as dying cells generate a burst of auxin that is transduced to the inner cell layers.^67^ A similar auxin burst may occur when p53 is induced in the epidermis and could account for the increased lateral root formation observed following recovery.

### Limitations of the study

Our research suggests recapitulation of p53 function across animals and plants. However, our findings do not directly elucidate the mechanisms by which p53 induces a developmental cell death-like program in plant systems. One hypothesis is that p53 transcriptional activity regulates the expression of genes involved in developmental cell death. However, we consider this is very unlikely due to the lack of promoter sequence conservation between plants and animals.^1^ We observe functional transferability at the level of protein-interactions and identified various processes that may contribute to the induction of PCD-like mechanisms. However, we do not explore the molecular meaning of the interactions further. While this mechanistic question is relevant and deserves further investigation, we recognize that given the critical importance of p53 in cancer research, it is imperative that we make our plant p53 expression system readily available to the broader research community and potentially help accelerate discoveries relevant to human health.

## Resource availability

### Lead contact

Further information and requests for resources and reagents should be directed to and will be fulfilled by the lead contact, Miguel de Lucas (miguel.de-lucas@durham.ac.uk).

### Materials availability

• Plasmids generated in this study have been deposited to Addgene.
• *Arabidopsis* lines generated in this will be provided upon request.

### Data and code availability

• Raw and processed proteomic data are available in the PRIDE repository under accession number PXD075858 (https://www.ebi.ac.uk/pride/archive/projects/PXD075858).
• This paper does not report original code.
• Any additional information required to re-analyse the data reported in this paper is available from the lead contact upon request.

## Acknowledgments

M.d.L. lab work is supported by seed corn funds from Durham University and by BBSRC Strategic LOLA (BB/V003534/1). P.M.’s lab is funded by DU start-up funds and the company Pleco Therapeutics. Y.L. was supported by the 10.13039/501100001809National Natural Science Foundation of China (grant no 32060663 X.W.) of China fellowship. C.C. was supported by the 10.13039/501100001809National Natural Science Foundation of China (32470578), the 10.13039/501100012166National Key Research and Development Program of China (2024YFE0102300), and the Fundamental Research Funds for the Central University (2662024SZ001) for funding support. J.E. is supported by a Durham University Doctoral Scholarship. A.A. is funded by CARA.

## Author contributions

Y.L. conducted experiments, data analysis, provided comments, and wrote the article. S.W. genotyped and identified XVE:p53 expressing plants. S.B. performed IP-MS analysis. J.E. performed PLACCI analysis. A.A. provided technical support. C.C. provided comments. X.W. provided resources. P.M. conceived the project, provided p53 gene resources, conceived experiments and wrote the article. M.d.L. conceived the project, conducted data analysis, and wrote the article. All authors read and approved the article.

## Declaration of interests

The authors declare that they have no known competing financial interests or personal relationships that could have appeared to influence the work reported in this paper.

## STAR★Methods

### Key resources table

**Table:** 

REAGENT or RESOURCE	SOURCE	IDENTIFIER
**Antibodies**		

anti-p53	Santa Cruz Biotechnology	Cat: sc-126; RRID: AB_628082
Protein A/G Magnetic Beads	Pierce	88802

**Bacterial and virus strains**		

*Escherichia coli*	Lucigen	60107
*Agrobacteria*: GV3101pSoup	N/A	N/A

**Biological samples**		

*Arabidopsis thaliana* – Columbia-0	N/A	N/A

**Chemicals, peptides, and recombinant proteins**		

MS salts	Duchefa	M0222
Tris-base	Melford Laboratories	T60040
MES	Duchefa	M1503
17β-estradiol	Merck Life Science	E8875
Agar	Melford Laboratories	A20020
Hygromycin	Duchefa	H0192
17β-estradiol	Melford Laboratories	E8875
Chloral Hydrate	Merck Life Sciences	15307
Propidium iodide	Melford Laboratories	P4170
Trypan blue	Thermo Fisher Scientific	15250061
diaminobenzidine	Merck Life Science	D12384
CuSO4	Merck Life Science	Merck Life Science
histo-ClearII	SLS	HS202
Formaldehyde	Merck Life Science	252549
Cellulase R-10	Duchefa	C8001
Driselase	Merck Life Science	D9515
Pectolyase y-23	Duchefa	P8004
DMSO	Thermo Fisher Scientific	
PMSF	Melford Laboratories	MB2001
DTT	Melford Laboratories	MB1008/

**Critical commercial assays**		

RevertAid RT	Thermo Scientific	10161310
HiFi Polymerase	PCR Bio	PB10.41

**Deposited data**		

Plant p53 pretein interactors	This paper. PRIDE repository	Table S1https://www.ebi.ac.uk/pride/archive/projects/PXD075858 PXD075858

**Experimental models: Organisms/strains**		

pWOL:GFP – Columbia-0	Birnbaum et al., 2003	pWOL:GFP
PlaCCi – Columbia-0	Desvoyes et al., 2020	PlaCCi
XVE:p53	This paper	
pDMP4:H2B:3xVenus	This paper	
pPASPA3:H2B:3xVenus	This paper	
pWOL:XVE:p53	This paper	
pWER:XVE:p53	This paper	

**Oligonucleotides**		

See Table S2 for a complete list of all oligonucleotides used in this study	IDT	N/A

**Recombinant DNA**		

pER8	Curtis and Grossniklaus, 2003	pER8
pMCY2	Emami, 2013	pMCY2

**Software and algorithms**		

ImageJ 1.44o	National Institute of Health	https://imagej.nih.gov/ij/
R	R Core Team (2021)	https://www.R-project.org/

### Experimental model and study participant details

#### Arabidopsis thaliana

All *Arabidopsis* plants used for this study were in Columbia-0 background. Transgenic plants expressing *XVE:p53, pWER:XVE:p53, pDMP4:H2B:3xVENUS* and *pPASPA3:H2B:3xVENUS* were generated via floral dip *Agrobacterium* transformation in Columbia-0 background.^68^ *pWOL:XVE:p53* transgenic plant was generated in pWOL:GFP background.

#### Escherichia coli

*E.coli* DH5α competent cells were used for routine molecular biology. All bacteria were grown in LB medium (Melford). The medium was also supplemented with 50 μg.ml^-1^spectinomycin (Melford) and/or 20 μg.ml^-1^streptomycin (Melford) as required to maintain the different plasmids.

### Method details

#### *Arabidopsis* growth conditions

Plants for propagation were grown under standard conditions at 21°C in a 16-h-light/8-h-dark cycle. For root analysis, *Arabidopsis* seeds were surface-sterilized and germinated on 1x Murashige and Skoog (MS) medium without sucrose. Seeds were stratified in dark for 3d at 4°C, then transferred into a Sanyo growth chamber with a PAR light intensity of 40μmol.m^-2^.s^-1^ illuminated by a daylight-white fluorescence lamp (FL40SS ENW/37; Panasonic) in a 24h-light cycle and 21°C of temperature. Seedlings were kept in vertical plates until analysis.

For all the experiments where the induction of the gene was performed via XVE system, sterile seeds were germinated on 1% MS media supplemented with 10 μM of 17ß-estradiol (Merck). The length of each treatment is indicated in the text and figure legends. Selection of transgenic seedlings was performed in 1% MS medium supplemented with 30 mg.ml^-1^ hygromycin or via mCherry seed fluorescence detection.^69^

#### Vector construction

For the construction of p53_pMDC7-1 plasmid, p53 coding sequence was amplified from a plasmid containing the human CDS of p53 using the primers p53_pMDC7_F and R and inserted into a XhoI and PacI digested pMDC7 plasmid through Hot Fusion.^70^ For the generation of the *pDMP4:H2B:3xVENUS* and *pPASPA3:H2B:3xVENUS* transcriptional reporter lines, the promoters of DMP4 and PASPA3 were amplified from genomic Col-0 DNA using the primers pDMP4_H2B_3xVenus_F and R, pPASPA3_H2B_3xVenus_F and R and inserted into a SapI digested vector pMCY2_H2B_3xVenus^71^ via Hot Fusion reaction.

For the construction of pWOL-XVE-p53 and pWER-XVE-p53, WOL and WER promoter sequences were amplified from plasmid p1R4-pWOL-XVE and p1R4-pWER-XVE using the primers pWOL-XVE-p53_F, pWER-XVE-p53_F and XVE-R BglII and inserted into the p53_pMDC7-1 plasmid digested with PmeI and BglII via Hot Fusion to create the final pWOL-XVE-p53 and pWER-XVE-p53 plasmids. All primers used in this study are listed in Data S2.

#### Root phenotypes

*Arabidopsis* roots were measured at 5-day-old. For ß-estradiol (Merck) treatments, the “6h” group was transferred 6 hours prior to the picture being taken on day 5; the “24h” group was transferred on day 4; and the “48h” group was transferred on day 3. Length measurements were done using ImageJ software.

#### CuSO4 treatment experiment

Five-day-old XVE:p53 seedlings were treated with DMSO (Thermo Fisher Scientific), 10 μM ß-estradiol and 10 μM ß-estradiol with different gradient concentration (0, 5, 10, 25 and 50 μM) CuSO4 (Merck) for 24h, respectively. Root length measurements were done using ImageJ software.

#### Recovery experiment

Three-day-old XVE:GUS, XVE:p53, pWOL:XVE:p53 and pWER:XVE:p53 seedlings were treated with DMSO and estradiol for 48h after germination, respectively. Then all the treated 5-day-old seedlings were moved to on 1x Murashige and Skoog (MS) medium (Duchefa) without sucrose for 5 days. Root length measurements were done using ImageJ software.

#### RNA extraction and qRT-PCR

To determine relative gene expression, roots of 5-day-old XVE:p53 seedlings treated with DMSO, 10 μM β-estradiol, or 10 μM β-estradiol with 25 μM CuSO_4_ for 24 h were collected. Root mRNA was extracted as described in Townsley et al.^72^ from three independent biological replicates, each consisting of approximately 100 roots. cDNA was synthesised using RevertAid reverse transcriptase (Invitrogen) according to the manufacturer’s instructions with oligo(dT) primers (IDT). qRT-PCR reactions were performed on a Rotor-Gene Q thermocycler (Qiagen) using gene-specific primers and a 3-step cycling protocol with an annealing temperature of 60°C and an extension time of 20 s. Differential expression was calculated from the mean Ct values of three biological replicates, each with three technical replicates. Amplification was normalised to the *PP2A* housekeeping gene (AT1G69960). Comparisons between two groups were made using Student’s t-test (∗*p* < 0.05, ∗∗*p* < 0.01, ∗∗∗*p* < 0.001, ∗∗∗∗*p* < 0.0001, ns = no significant). Comparisons between multiple groups were made using ANOVA + Tukey HSD test (*p* < 0.05). Primer sequences used for transcript detection are listed in Data S2.

#### Confocal analysis

T3 homozygous plants (validated by antibiotic selection) carrying the XVE:p53 transgene alone or in combination with transcriptional reporters for developmental cell death markers or cell cycle phases were imaged 5 days after germination following the treatments described above. Imaging was performed on Zeiss LSM 800 and 880 confocal microscopes equipped with a 20x (NA 0.8) and 40x (NA 1.3) objectives, Airyscan detection, and sequential scanning with automated merging (Department of Biosciences, Durham University) using Zen software. Excitation and detection parameters were set as follows: Venus/YFP, excitation at 488 nm and detection at 499–571 nm; GFP and alexa-fluor 488, excitation at 488 nm and detection at 493–558 nm; CFP, excitation at 422 nm and detection at 452–505 nm; mCherry, excitation at 561 nm and detection at 580–650 nm; PI, excitation at 561 nm and detection at 605–695 nm. All signals were confirmed across multiple independent roots (*n* > 5 per experiment). A representative image of each fluorescence signal is shown in the corresponding figure.

#### Histochemical staining

DAB staining was performed as described in.^24^^,^^73^ Five-day-old XVE:p53 seedlings treated with DMSO, ß-estradiol, and ß-estradiol with different gradient concentration CuSO_4_ for 24h were incubated in DAB solution(0.5 mg 3, 3′-diaminobenzidine, 50 mM Tris acetate buffer pH 3.8, Melford) freshly prepared in the dark for 18 h at 25°C. Stained samples were then bleached using a solution of ethanol/acetic acid/glycerol at a 3:1:1 ratio. Finally, pictures of stained tissues were captured using a Nikon Stereozoom microscope (Nikon, Tokyo, Japan). Trypan blue staining (Thermo Fisher Scientific) was performed as previously described.^23^ Seedlings were covered with trypan blue solution (30 mL of ethanol, 10 g of phenol, 10 mL of water, 10 mL of glycerol, 10 mL of lactic acid, and 10 mg of trypan blue), placed in a boiling water bath for 2 to 3 min, and then left at room temperature for 1 hr. The samples were transferred into a chloral hydrate solution (2.5 g/mL, Merck) and boiled for 20 min to destain. After multiple exchanges of chloral hydrate solution to reduce the background, samples were equilibrated with 50% glycerol, mounted, and observed with a stereomicroscope (Nikon, Tokyo, Japan).

#### Whole-mount immunolocalization

Whole mount immunolocalization was carried out as previously described.^74^ Five-day-old XVE:p53 seedlings were harvested after being treated with DMSO and β-estradiol for 24 h. Fix the collected seedlings in fresh BVO buffer (1x PBS, 2 mM EGTA pH 7.5, 1% Formaldehyde, 10% DMSO, 0.1% Tween, Melford) for 30 mins at room temperature. Removing fixed seedlings to PBT buffer (1x PBS and 0.1% Tween) on ice. Embedding seedlings into acryl gel (24 ml 10 % APS, 12 ml TEMED, 5 % acrylamide, Biorad) on slides and cover with coverslips at room temperature for 1h and then remove the coverslip after the acryl gel is dry. Putting all of slides with seedlings in Coplin jars and treating samples with corresponding reagents (methanol for 5mins, ethanol for 5mins, histo-ClearⅡfor 30 mins, ethanol for 5mins, methanol for 5mins, methanol:PBT (1:1) and 2.5% Formaldehyde for 15 mins). Next, rinse samples with PBT buffer for 10mins at twice. For digesting the cell wall with 0.5% cellulase (Duchefa), 1% driselase (Merck), 0.5% pectolyase (Duchefa) in PBS buffer at 37°C for 2h and then wash samples with PBT buffer for 5mins at twice. Incubating each slide with 100 ml of RNAseA at 100 mg/ml in PBS with 1% Tween-20 for 1 hr at 37°C and wash with PBT buffer for 5 mins at twice. Then, post fix samples with fresh PBT-F buffer for 20 minutes and wash with PBT buffer for 10 minutes. Finally washing samples with PBT for 5 minutes twice after incubating seedlings with PBS and 2%Tween-20 shaking for 2h at 4°C.

For immunodetection, incubating each slide with 100 ml of anti-p53 (FL393, Santa Cruz, 0.2 μg/ml) p53 DO-1 primary antibody (FL393, Santa Cruz, 0.2 μg/ml) diluted in PBS with 0.2% Tween-20 for overnight at 4°C, and washing 2h in PBT buffer at room temperature before applying the secondary antibody anti-mouse Alexa Fluor 488 (Life Technologies) 1:200 in PBS + 0.2% Tween-20 for overnight at 4°C, washing 1h in PBT buffer again. Finally, counterstain samples with 10 μg/ml propidium iodide in PBS for 15 min, then rinse 15 min in PBS under gentle shaking, at room temperature. Representative fluorescent images were obtained using a Zeiss 800 confocal microscope (Zeiss 800, Durham, UK) after mounting samples in Prolong Gold (Invitrogen) with 5 mg/ml propidium iodide.

#### P53 immunoprecipitation and IP-MS analysis

Transgenic XVE: p53 seeds were germinated in liquid Gamborg B5 medium (Duchefa) supplemented with B5 vitamins and sucrose and grown under constant agitation for three weeks in the dark to promote root growth. To induce p53 expression, β-estradiol was added to the growth medium at a final concentration of 10 μM 24 hours prior to harvesting; an equivalent volume of DMSO was added to control samples. Harvested tissue was blotted dry, immediately snap-frozen in liquid nitrogen, and stored at −80°C until further processing. For protein extraction, frozen tissue was ground to a fine powder using a pre-chilled mortar and pestle and resuspended in extraction buffer containing 50 mM Tris-HCl (pH 7.5), 150 mM NaCl, 0.5% Triton X-100, 1 mM PMSF, and a protease inhibitor cocktail. Crude extracts were clarified by centrifugation, and the resulting supernatant was used as input for immunoprecipitation (IP).

For the IP, Dynabeads sheep anti-mouse IgG (Thermo Fisher Scientific) were washed 3 x in extraction buffer without the triton and PMSF and then pre-coupled tumbling for 1 h at 4°C with anti-p53 DO-1 (2 μg per IP, Santa Cruz). Lysates were added to the beads and incubated overnight at 4°C. After this the beads were washed with ammonium bicarbonate (ABC) to remove detergent and unspecific binders. The proteins were then reduced by 100 mM DTT for 30 min, followed by alkylating with iodoacetamide for 30 min in the dark. The samples were trypsinised (Promega) at 37°C overnight at 700 rpm. The beads were washed with acetonitrile (ACN). After the peptides were eluted with 2% DMSO and ACN. This was diluted with 0.1% formic acid (FA) until the DMSO concentration reached <1%, this was loaded on the mass spectrometer.

#### MS running and further processing

IP-digest eluates were dried, and the residue resuspended in 0.5% formic acid (FA) for desalting using C18 Stage tips. Resulting peptide residues were resuspended in 0.1% FA for top-30 data-dependent LC-MS-MS analysis on a TripleTOF 6600 mass spectrometer linked to an Eksigent 425 LC via a DuoSpray 2 source (Sciex). Sample injection and peptide separation was in trap and elute mode using YMC-TriArt C18 columns (trap, part no. TA12S05-E5J0RU, resolving TA12S03-15H0RU) at a flow rate of 0.5 μL/min. Buffer A was 0.1% FA in water and B 0.1% FA in acetonitrile and peptides eluted in sequential gradients of: 3 - 8% B over 3 minutes, 8 – 30% B over 35’, 30-40 % B over 9 minutes, followed by a 2 minute ramp to 80 % B and a column wash for 5 minutes before re-equilibration in 3% buffer B.

MS data acquisition was for 50.5 minutes after valve switching to bring the trap in-line and the mass spectrometer cycle time of 1.2 seconds comprised a 0.25 sec precursor MS1 scan (350 – 1600 m/z) followed by 30 MS-MS acquisitions of 30 msec (100 – 1500 m/z range). MS-MS was triggered by precursors of +2 to +5 charge with >500 cps intensity and a rolling exclusion of 15 seconds was applied throughout to limit selection of the same precursor multiple times.

Datafiles in wiff format were processed using PEAKS X-Pro (Bioinformatics Solutions Inc) using a database containing the Arabidopsis thaliana proteome, known proteomic experiment contaminants and the human p53 protein sequence, a total of 33,781 entries. Amino acid modifications were fixed carbamidomethyl [C] and variable oxidised methionine. Post-processing, the peptide FDR was set to 1% against a reversed decoy database, the peptide *de novo* ALC set to >80%, protein FDR set to 1% and a filter of 2 unique peptides per protein was applied. Identified protein and protein-peptide lists were exported from PEAKS for further analysis. Raw and processed proteomic data are available in the PRIDE repository (https://www.ebi.ac.uk/pride/) under accession number PXD075858.

#### Western blot

For analysing the protein expression of p53 after induction in *Arabidopsis* roots, five-day-old seedlings of the XVE:p53 treated with DMSO and ß-estradiol for 6h, 24h and 72h, respectively. And for exploring p53 protein expression after CuSO4 treatment, five-day-old seedlings of the XVE:p53 were treated with ß-estradiol, ß-estradiol with 25 μM CuSO4, and ß-estradiol with 50 μM CuSO4 for 24h, respectively. All samples were collected for protein extraction (1X PBS, 0.1% Tween, 0.1% SDS, 2% β-mercaptoethanol, 1 mM PMSF, 1× EDTA-Free Protease Inhibitor Cocktail and centrifuge samples for 15 minutes maximum speed 4°C. Transferring the supernatant into new tube and measuring protein content with Bradford assay (Bio-Rad). Equal protein was diluted with protein loading dye with 10% β-mercaptoethanol (Merck) and boiled at 98 °C for 10 mins. Then samples were separated in 10% SDS-PAGE and transferred onto nitrocellulose membranes. After blocking with 10% skimmed milk in TBST for 1h at RT, the membranes were incubated with primary antibodies anti-p53 (FL393, Santa Cruz, 0.2 μg/ml) in TBST. The blots were washed three times with TBST and then incubated with IRDye anti-mouse secondary antibody 800CW (Li-Cor, 0.05 μg/ml). After three times washing with TBST, protein signals were visualized using the ODYSSEY Sa infrared system (Li-Cor, Cambridge, UK) and Image Studio software 2.0 (Li-Cor).

#### Quantification analysis

Fluorescent intensity was measured in the root stele by Image J. The intensity value of estradiol treatment was relative to DMSO treatment, and the DMSO treatment value was summarised to ‘1’. Trypan blue images were split into 3 channels, and the blue channel image was measured before setting threshold by Image J. DAB staining images were also split into 3 channels, and the brown channel image was before setting threshold and calibrating OD by Image J.

### Quantification and statistical analysis

Statistical parameters including N and statistical significance are reported in the figures and figure legends. N represents the number of seedlings in Figures 1G, 3B, 4C, 4E, 4G, 4I, S2B and S4B.The statistical significance of the data was tested in GraphPad Prism version 10. (GraphPad Software Inc., San Diego, CA, USA: http://www.graphpad.com/). Comparisons between two groups were made using Student’s t-test (∗*p* < 0.05, ∗∗*p* < 0.01, ∗∗∗*p* < 0.001, ∗∗∗∗*p* < 0.0001, ns = no significant). Comparisons between multiple groups were made using ANOVA + Tukey HSD test (*p* < 0.05).

## Supplemental information

Document S1. Figures S1–S6 and Data S3

Data S1. Lists of the proteins identified by IP-MS in each of the three biological replicates of the control and p53-induced root cultures (related to Figure 2E), along with the Gene Ontology terms associated with their biological function

Data S2. List of the oligonucleotides used in this study
