# Supplementary material for: A versatile inducible plant model system for dissecting p53 function and discovering novel interaction networks
Source: iScience. 2026 Jul 14;29(8):116775. doi: 10.1016/j.isci.2026.116775 (PMC13382806; doi:10.1016/j.isci.2026.116775)

iScience, Volume *29*

Supplemental information

A versatile inducible plant model system for dissecting p53 function and discovering novel interaction networks

Yuqi Li, Shuyuan Wang, Steven Bell, Joanna Edwards, Ahmed Alboraey, Xiaopeng Wen, Chunli Chen, Patricia Muller, and Miguel de Lucas

SUPPLEMENTARY FIGURES:

Figure S1:

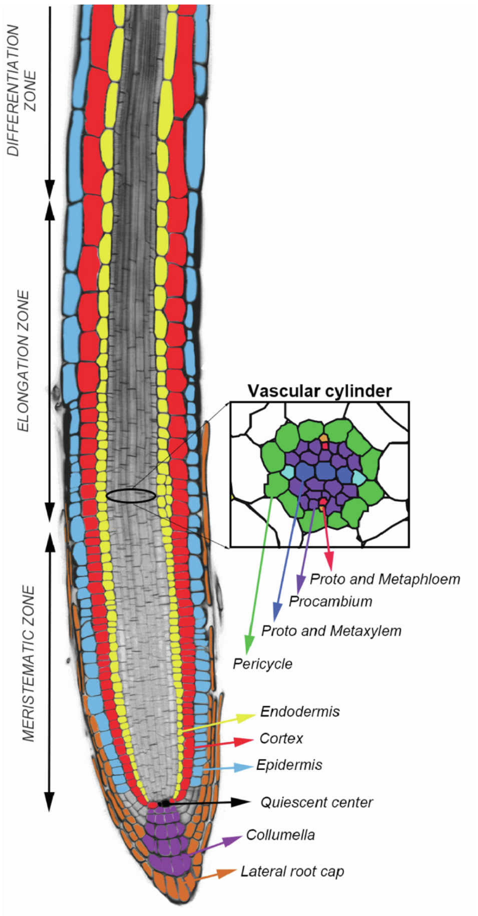

Figure S1: *Arabidopsis* root structure

Figure S2:

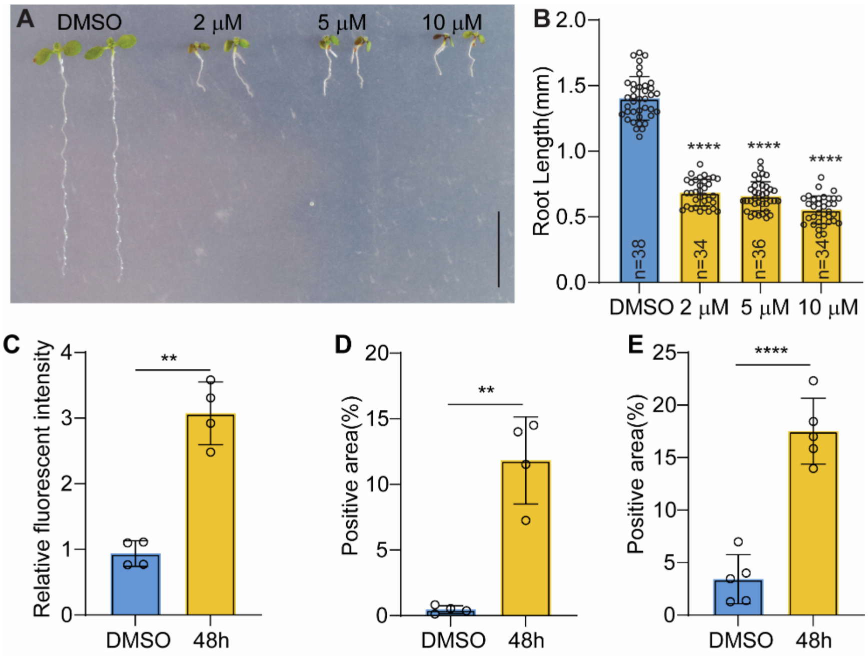

Figure S2: Effect of ß-estradiol concentrations on p53 induction.

(A) Representative pictures of 7-day-old XVE:P53 seedlings treated with DMSO and gradient concentration estradiol for 4 days. Scale bar, 0.5 cm. (B) Primary root length measurements. N is number of seedlings, Bar plots represent the mean ± SD and asterisks indicate significant differences between samples (****, p < 0.0001 by Student's t-test). (C)Fluorescent intensity of PI staining cells in the root stele. (D) The percentage area of trypan blue staining cells in XVE:p53 roots treated with DMSO and estradiol. (E) The percentage area of DAB staining cells in XVE:p53 roots treated with DMSO and estradiol. ((*, P < 0.05; ****, P < 0.0001 by Student's t-test).

Figure S3:

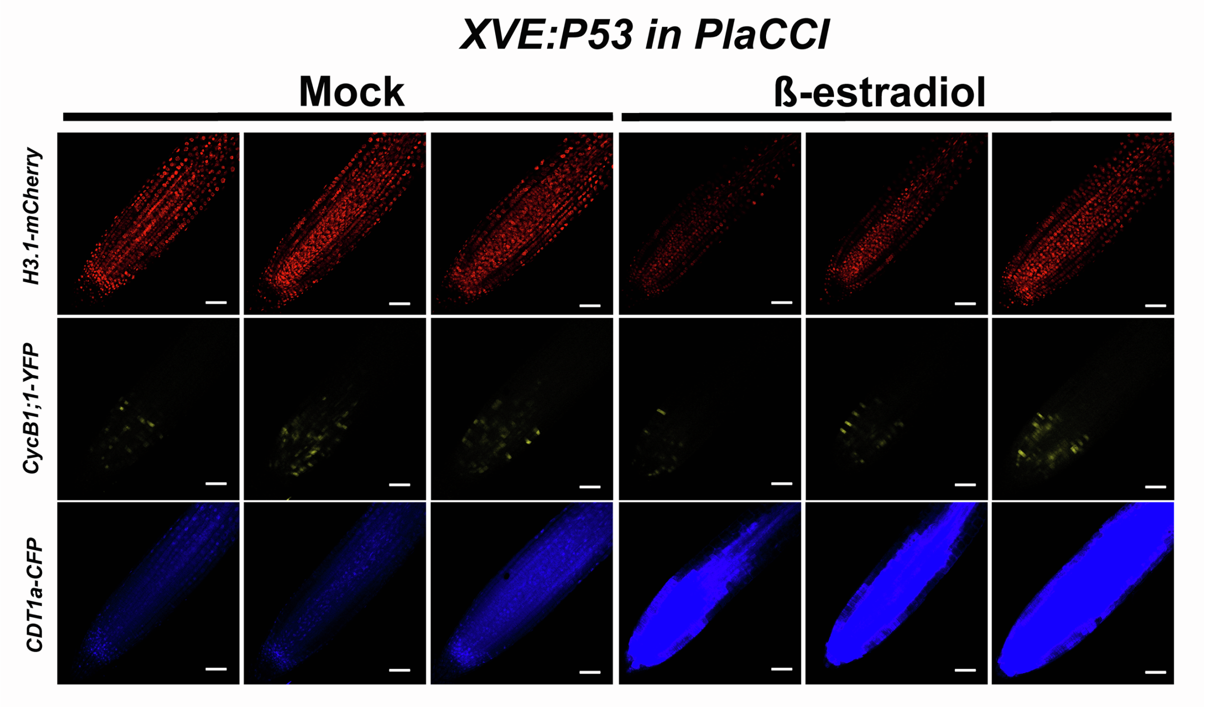

Figure S3: Root meristem confocal images of 5-day old seedlings expressing the cell cycle marker PlaCCI in the XVE:p53 background. Mock and ß-estradiol-treated roots for 24h. Scale bar, 50 µm.

Figure S4:

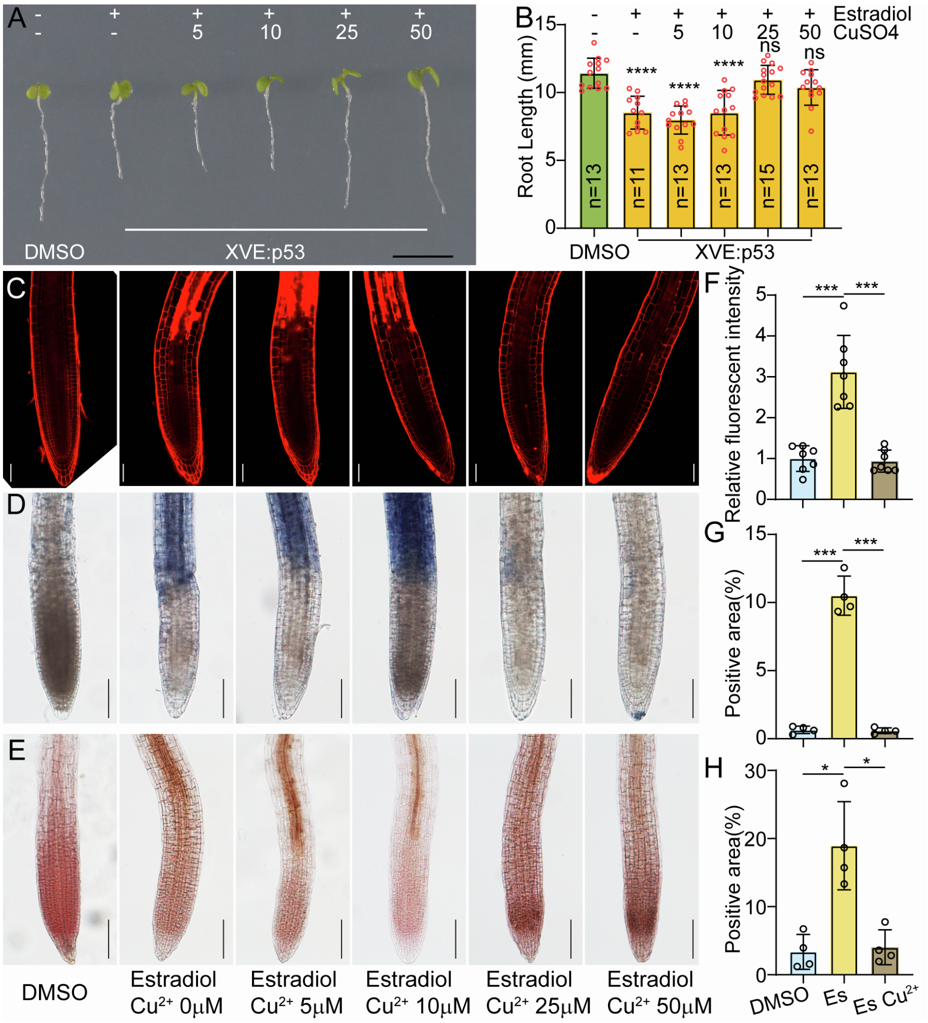

Figure S4: Copper concentration impact on p53 inhibiting root growth

(A) Representative 5-day-old XVE:p53 root seedlings treated with DMSO, 10 μM estradiol and gradient concentration $CuSO_4$, respectively. Scale bar, 0.5_cm. (B) Primary root length measurements of 5-day-old XVE:p53 seedlings treated with DMSO, 10 μM estradiol and gradient concentration $CuSO_4$, respectively. N is the number of seedlings, Bar plots represent the mean ± SD and asterisks indicate significant differences between samples (****, $p < 0.0001$, ns, no significant by Student's t-test). (C–E) Representative images of 5-day-old XVE:p53 roots treated with DMSO, 10 μM ß-estradiol, or 10 μM ß-estradiol supplemented with increasing concentrations of $CuSO_4$ (5, 10, 25 and 50 μM), from left to right. (C) Confocal images of roots stained with propidium iodide. Scale bar, 50 μm. (D) Trypan blue staining for the detection of cell death. Scale bar, 200 μm. (E) Accumulation of H_2O_2 detected by DAB staining. Scale bar, 200 μm. (F) Fluorescent intensity of PI staining cells in XVE:p53 roots treated with DMSO, estradiol and estradiol with $CuSO_4$. (G) The percentage area of trypan blue staining cells in XVE:p53 roots treated with DMSO, estradiol and estradiol with $CuSO_4$. (H) The percentage area of DAB staining cells in XVE:p53 roots treated with DMSO, estradiol and estradiol with $CuSO_4$. All the 5-day-old XVE:p53 seedlings were treated with DMSO(Mock), 10 μM ß-estradiol(Es) and 10 μM ß-estradiol with 25 μM $CuSO_4$ (Es Cu^{2+}) for 24h, respectively. (*, $P < 0.05$; ***, $P < 0.001$ by Student's t-test).

Figure S5:

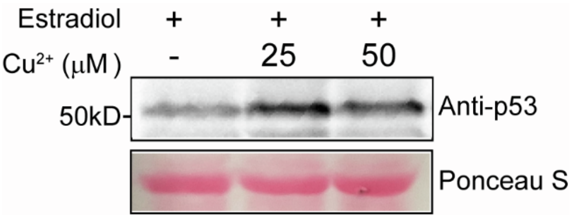

Figure S5: p53 protein level after $CuSO_4$ treatment

Figure S6:

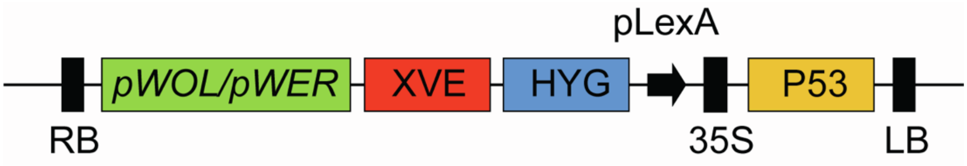

Figure S6: Construction of the inducible expression vector with p53 by the XVE vector with specific promoter.

DATA S3: UNCROPPED BLOTS

Ponceau staining and p53 protein detection images associated with the data presented in Figure 1C and Figure S5

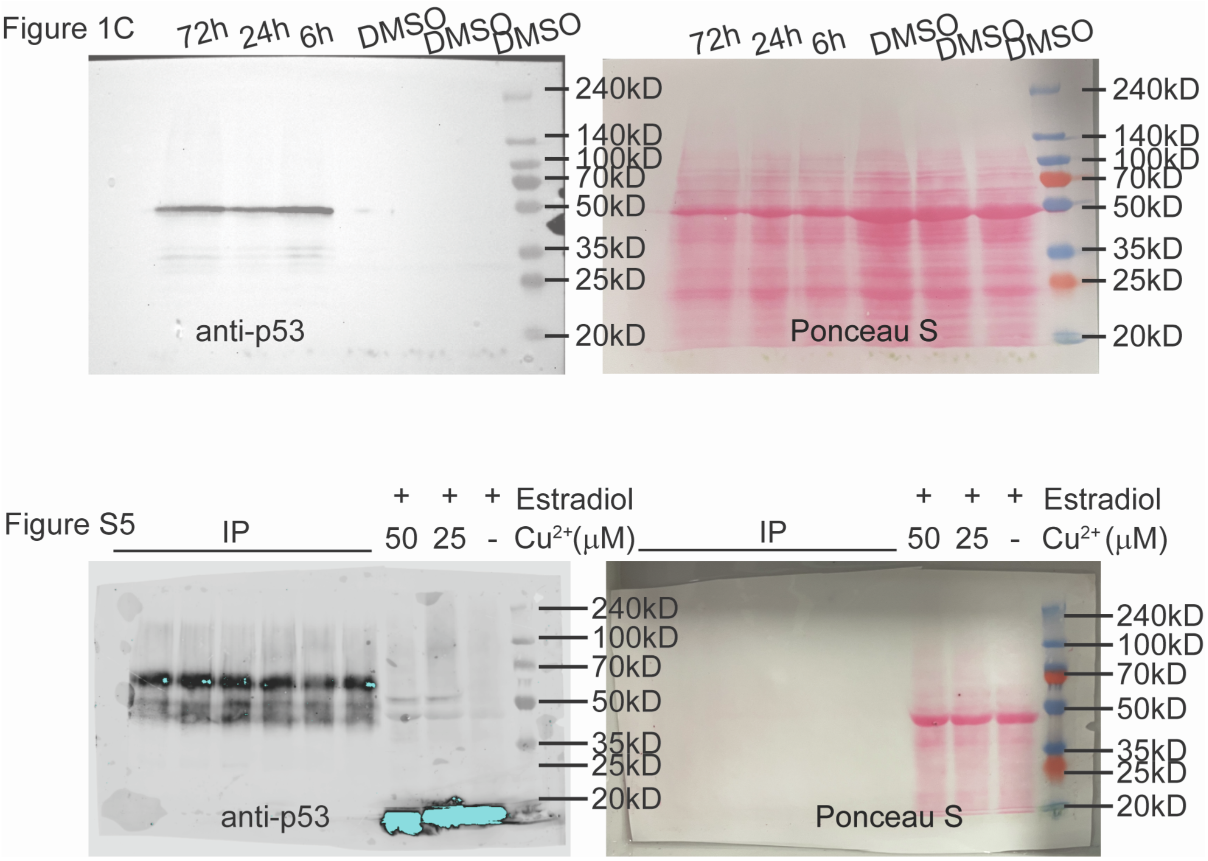

Supplement: Document S1. Figures S1–S6 and Data S3 [file mmc1.pdf]
